# Model of leaf biomass partitioning coefficient in different main stem leaf ranks of rapeseed (*Brassica napus* L.)

**DOI:** 10.1371/journal.pone.0330011

**Published:** 2026-02-05

**Authors:** Weixin Zhang, Wenyu Zhang, Qian Wu, Chuanliang Sun, Daokuo Ge, Jing Cao, Hong Li, Hongxin Cao

**Affiliations:** 1 Research Center of Fluid Machinery Engineering and Technology, Jiangsu University, Zhenjiang, Jiangsu, P.R. China; 2 Institute of Agricultural Information/ IGRB-IAI Joint Laboratory of Germplasm Resources Innovation & Information Utilization, Jiangsu Academy of Agricultural Sciences, Nanjing, Jiangsu, P.R. China; Canakkale Onsekiz Mart University, TÜRKIYE

## Abstract

Leaf growth is a dynamic process that critically determines canopy architecture and assimilate allocation in rapeseed. Quantifying the distribution of leaf biomass along the main stem across developmental stages is essential for advancing functional-structural plant models of rapeseed. To address the lack of a leaf biomass partitioning model in existing rapeseed growth models, this study developed a rank-specific leaf biomass partitioning coefficient model for the main stem in rapeseed. Field experiments were conducted over three growing seasons (2012–2015) using three cultivars: Ningyou 18 (V1, conventional), Ningyou 16 (V2, conventional), and Ningza 19 (V3, hybrid). The experiments were conducted under factorial combinations of cultivar, nitrogen fertilizer, and transplanting density. The leaf biomass partitioning coefficient was calculated as the ratio of leaf biomass at a given leaf rank to the total main-stem leaf biomass, with leaf ranks normalized to the (0–1] interval. Model parameters were estimated to elucidate how cultivar and environmental factors influence partitioning patterns across leaf positions. Validation using independent experimental data showed strong agreement between the simulated and observed values, with a correlation coefficient (*r*) more than 0.9 (*p* < 0.001). The mean absolute difference (*d*_*a*_) ranged from −0.080 to 0.011 g g^-1^, and the ratio of *d*_*a*_ to the average observation (*d*_*ap*_) varied between 3.077% (anthesis stage) and 13.083% (normalized leaf rank). The root mean square error (*RMSE*) values were all below 0.193 g g^-1^ across all stages, with the most stage-specific *RMSE* values under 0.032 g g^-1^. The results demonstrate that the model performs reliably in simulating the main-stem leaf biomass partitioning coefficient across hierarchical leaf ranks in rapeseed. By integrating leaf-level biomass allocation with whole-plant growth processes, this work provides a key component for developing a functional-structural rapeseed model and supports further research on source-sink regulation and canopy optimization.

## Introduction

As principal photosynthetic organs, leaves perform critical physiological processes including light energy conversion, gaseous exchange, and photoassimilate production through coordinated photosynthetic and respiratory activities [1]. The ontogeny of leaves initiates through leaf primordia formation, a developmental process exhibiting phyllotactic coordination with branch, floral bud, and pod organogenesis [2]. Empirical evidence demonstrates that photosynthetic enhancement induces carbohydrate surplus, creating positive feedback mechanisms that stimulate leaf morphogenesis [3]. In *Brassica napus*, the architectural configuration of plants is fundamentally determined by leaf morphological characteristics, phyllotaxis patterns, and population density. Strategic optimization of foliar quantity and biomass distribution patterns presents opportunities for improving canopy architecture, augmenting photosynthetic performance, rationalizing biomass allocation, and ultimately enhancing crop productivity and quality.

Contemporary understanding of biomass allocation mechanisms reveals that assimilate partitioning among plant organs critically influences growth dynamics and yield formation [4]. Traditional allometric models, despite their widespread adoption in crop simulation platforms, employ static organ growth ratios that constrain predictive accuracy to specific environmental regimes [5]. While providing reasonable approximations under controlled conditions, these empirically-derived approaches inadequately capture the dynamic reallocation patterns observed under fluctuating resource availability [6]. More critically, their inherent empirical nature fails to explain emerging evidence of carbon-nitrogen stoichiometric regulation in biomass partitioning [7,8]. Consequently, quantitative and mechanistic insights into biomass allocation are often limited. While these models may closely align with experimental results, they often describe biomass distribution within a narrow range of growth conditions and fail to account for dynamic fluctuations in biomass allocation [9].

This conceptual limitation has driven the development of hybrid modelling paradigms. Canonical modelling approaches attempt to bridge empirical and mechanistic extremes by incorporating semi-quantitative physiological principles [10], enabling simulation of complex allocation dynamics without exhaustive parameterization. Recent advances in crop simulation further highlight the importance of accounting for intra‑canopy physiological heterogeneity. For example, Shin et al. demonstrated in cucumber that leaf‑position‑specific photosynthetic models significantly improve the accuracy of dry matter partitioning predictions compared to uniform canopy representations [11]. This underscores the functional relevance of leaf rank and developmental stage in determining photosynthetic capacity and assimilate supply—a dimension often overlooked in traditional whole‑canopy models.

Validation studies reveal that sink strength modulation, defined as the organ-specific growth potential under non-limiting assimilate supply-exerts predominant control over partitioning patterns [12]. Quantitative characterization of sink strength, defined as the maximum potential growth rate under non-limiting assimilate supply, provides a physiologically grounded framework for modelling inter-organ allocation dynamics [13,14]. The transport resistance model [15–18] offers a detailed mechanistic representation of assimilate flux from source to sink tissues. However, implementation challenges arising from parameterization complexity and limited operational impact assessments have restricted its adoption in production-scale crop models.

Notably, a critical knowledge gap persists regarding leaf biomass partitioning along ontogenetic gradients in rapeseed (*Brassica napus* L.). Current models predominantly address organ-level allocation rather than intra-organ gradients, despite evidence that leaf rank position influences both photosynthetic competence and senescence patterns. As illustrated in studies on cucumber and other crops [11], incorporating leaf‑position‑specific physiological traits can substantially enhance the realism and predictive power of partitioning models. Precise characterization of main stem leaf biomass distribution represents an essential step toward understanding whole-plant resource optimization strategies, particularly for improving stress resilience and carbon sequestration efficiency in breeding programs.

Building upon our previous work in rapeseed organ-level partitioning [19], this investigation establishes a novel framework for modeling main stem leaf biomass distribution across developmental ranks. Our approach integrates functional-structural plant model principles with sink strength dynamics, aiming to advance precision agriculture applications through improved canopy architecture optimization and resource use efficiency prediction in rapeseed cultivation systems.

## Materials and methods

### Materials

Three rapeseed (*Brassica napus* L.) cultivars were used: ‘Ningyou 18’ (V1, conventional), ‘Ningyou 16’ (V2, conventional), and ‘Ningza 19’ (V3, hybrid). All cultivars were developed by the Institute of Economic Crops Research of Jiangsu Academy of Agricultural Sciences.

### Ethics statement

This study did not involve any human or animal subjects. Therefore, ethical approval or informed consent was not required.

### Methods

#### Experimental site and conditions.

Field experiments were conducted during four consecutive rapeseed growing seasons (2012−2015) at the experimental farm of Jiangsu Academy of Agricultural Sciences, Nanjing, China (32.03°N, 118.87°E). The site is a rainfed site, with an average annual rainfall of 1106.5 mm and a relative humidity of 76%. Under well-drained field conditions, rapeseed did not experience water stress, and no supplemental irrigation was applied. The soil is classified as a hydraulic anthrosol, with the following initial properties: organic carbon, 31.4 g kg^-1^; total nitrogen, 2.03 g kg^-1^; available phosphorus, 20.3 mg kg^−1^; available potassium, 139.0 mg kg^−1^; and pH 7.31.

#### Experimental design.

Four separate experiments were carried out over consecutive growing seasons to evaluate the effects of cultivar, nitrogen (N) fertilizer rate, and transplanting density on rapeseed growth. The specific design for each experiment is summarized in Table 1.

**Table 1:** Overview of experimental designs across growing seasons.

Year	Experimental factors	Cultivars	N levels (kg N ha^-1^)	Density levels (plants ha^-1^)
2012-2013	Cultivar × Fertilizer × Density	V1, V3	N0 = 0; N1 = 90; N2 = 180	D1 = 6 × 10 ^4^; D2 = 12 × 10^4^; D3 = 18 × 10^4^
2013-2014	Fertilizer × Density	V1	N0 = 0; N1 = 90; N2 = 180	D1 = 6 × 10 ^4^; D2 = 12 × 10^4^; D3 = 18 × 10^4^
2013-2014	Cultivar	V1, V2, V3	N1 = 90	D2 = 12 × 10^4^
2014-2015	Cultivar × Fertilizer	V1, V3	N0 = 0; N1 = 90; N2 = 180; N3 = 270; N4 = 360	D2 = 12 × 10^4^

**Experiment 1 (2012–2013): cultivar × fertilizer × density:** A split-plot design with three replications was used. Whole-plot treatments consisted of three N fertilizer levels: N0 (0 kg N ha ^−1^), N1 (90 kg N ha ^−1^), and N2 (180 kg N ha^−1^). Sub-plot treatments comprised two cultivars (V1: ‘Ningyou 18’, and V3: ‘Ningza 19’) and three transplanting densities: D1 (6 × 10 ^4^ plants ha^−1^), D2 (12 × 10^4^ plants ha^−1^), and D3 (18 × 10^4^ plants ha^−1^).

**Experiment 2 (2013 − 2014): fertilizer × density:** A split-plot design with three replications was employed. Whole plots were assigned three N fertilizer levels (N0, N1, N2), and sub-plots included one cultivar (V1: ‘Ningyou 18’) and three transplanting densities (D1, D2, D3).

**Experiment 3 (2013–2014): cultivar comparison:** A randomized complete block design with three replications was used to compare three cultivars (V1, V2, V3) under uniform fertilization (N1: 90 kg N ha ^−1^) and density D2 (12 × 10^4^ plants ha^−1^).

**Experiment 4 (2014–2015): cultivar × fertilizer:** A split-plot design with three replications was applied. Whole-plot treatments included five N fertilizer levels: N0 (0 kg N ha^−1^), N1 (90 kg N ha^−1^), N2 (180 kg N ha^−1^), N3 (270 kg N ha^−1^), and N4 (360 kg N ha^−1^). Sub-plots consisted of two cultivars (V1 and V3). Transplanting density was fixed at D2 (12 × 10^4^ plants ha^-1^).

All plots measured 3.99 m × 3.5 m, with row spacing of 0.42 m. Plant spacing was adjusted according to the target density. Phosphorus (as P₂O₅) and potassium (as K₂O) were applied at rates equivalent to the N level (e.g., 90 kg ha ⁻ ¹ each for N1, 180 kg ha ⁻ ¹ each for N2, etc.). Boron (15 kg ha ⁻ ¹) was foliar-applied at bolting. Basal and top-dressing N were split in a 5:3:2 ratio at wintering and bolting stages. Sowing dates were 15 Oct 2011, 8 Oct 2012, 4 Oct 2013, and 4 Oct 2014; transplanting dates were 4 Nov 2011, 9 Nov 2012, 1 Nov 2013, and 1 Nov 2014. Field management (ditching, weeding, pest control, etc.) followed local high-yield practices.

### Sampling and measurements

Prior to transplanting, the leaf rank (i.e., ordinal position on the main stem) was marked on 50 randomly selected seedlings per plot using a number stamp. Destructive sampling was conducted at least once per growth stage, with three to five plants collected per treatment. The plant height and the height of each leaf rank were recorded. Leaves, stems, flower buds, flowers, and pods from the main stem and primary/secondary branches were separated by organ and leaf rank. All samples were oven-dried at 105 $\backslash {(}^{\circ }\text{C}$ for 30 min, then at 80 $\backslash {(}^{\circ }\text{C}$ to constant weight, and dry weight was measured using a 0.001-g electronic balance.

#### Calculations and statistical analysis.

The day of physiological development (*DPD*), used to drive the model [20], was calculated as follows:


$$DPD\;=\sum_{i=\text{1}}^{N}e^{kj}.{T_{ebj}}^{pj}.{T_{euj}}^{qj}.{P_{ej}}^{Gj}.f(E_{Ci})$$ (1)

Where the *N* is the number of days covering the stage, the *kj* is a basic development parameter which is determined by cultivar heredity, the *T*_*ebj*_ and the *T*_*euj*_ are the effective factors for lower and upper temperature, respectively, the *pj* and the *qj* are the genotypic coefficients of temperature effects, the *P*_*ej*_ is the effective factor of photoperiod, *Gj* is the genotypic coefficient of photoperiod effects, and *f*(*E*_*Ci*_) is the effective function of agronomic practice factors for rapeseed. All the symbols “*j*” above mean at the *j*th stage. The calculation of *DPD* in each stage, such as the effects of photoperiod and vernalization, has been briefly described in Appendix A. More details were explained in our existing rapeseed phenology model [19,21,22]. We normalized *DPD* into the [0, 1] interval to calculate and express easily. Normalized *DPD* (**n*DPD*) values of 0 and 1 correspond to sowing and physiological maturity, respectively.

For clarity, the developmental stages were categorized based on **n*DPD* ranges as follows:

Earlier stage (seedling-budding): **n*DPD* = 0–0.428, corresponding to the initial growth phase from emergence to the onset of rapid vegetative growth.

Mid stage (budding-early blooming): *nDPD* = 0.428–0.640, representing the period of active stem elongation and branching.

Late stage (early blooming-mature): *nDPD* = 0.640–1, covering reproductive development from flowering to maturity.

These thresholds were established based on phenological observations from preliminary studies and were used consistently across all analyses.

Biomass partitioning coefficient of leaf (*PC*_*L*_) is defined as follows:


$$PCL\;(LR)\;=\;\frac{{Bio}_{L}(i)}{{Bio}_{TL}}$$ (2)

where *Bio*_*L*_ (*i*) is the biomass of the *i*th leaf at the main-stem, and *Bio*_*TL*_ is the total biomass of the main-stem leaf.

To account for differences in total leaf number and facilitate modelling, the measured leaf rank in each treatment was divided by the total leaf number to obtain a normalized leaf rank within the (0–1] interval.

Data from the 2013–2014 growing season were used for model development, while data from the 2012–2013 and 2014–2015 growing seasons were employed for model validation. A multi-way analysis of variance (ANOVA) was conducted to examine differences in key morphological traits, which include plant height, and the height of the maximum leaf rank, and the biomass of green leaves and total leaves on the main stem, across different experimental treatments. Experimental data were organized and preprocessed using Microsoft Excel 2016, with subsequent grouped data fitting and selection of the optimal fitting equation form performed via R software (version 4.5.1); model performance was evaluated based on standard deviation and correlation coefficient.

#### Model validation.

The models developed in this paper were validated by calculating the correlation (*r*), the root mean square error (*RMSE*), the average absolute difference (*d*_*a*_), and the ratio of da to the average observation (*d*_*ap*_) [23], and a 1:1 line of simulated and observed properties. Some statistical indices are defined as follows:


$$RMSE=\sqrt{\frac{\sum_{i=\text{1}}^{n}{\left(O_{i}-S_{i}\right)}^{\text{2}}}{n}}$$ (3)

$$d_{a}=\frac{\text{1}}{n}\sum_{i=\text{1}}^{n}\left(O_{i}-S_{i}\right)$$ (4)

$$d_{ap}=\frac{\left|d_{a}\right|\;}{\overset{―}{O}}\times \text{1}\text{0}\text{0}\%$$ (5)

where *i* is the sample numbers, *n* is the total number of measurements, **n* *=* *n**-1 when n ≥ 30, *S*_*i*_ is the simulated value, and *O*_*i*_ is the observed value.

## Results

### Variance analysis of leaf biomass partitioning coefficient and green leaf numbers

**Fig 1:**
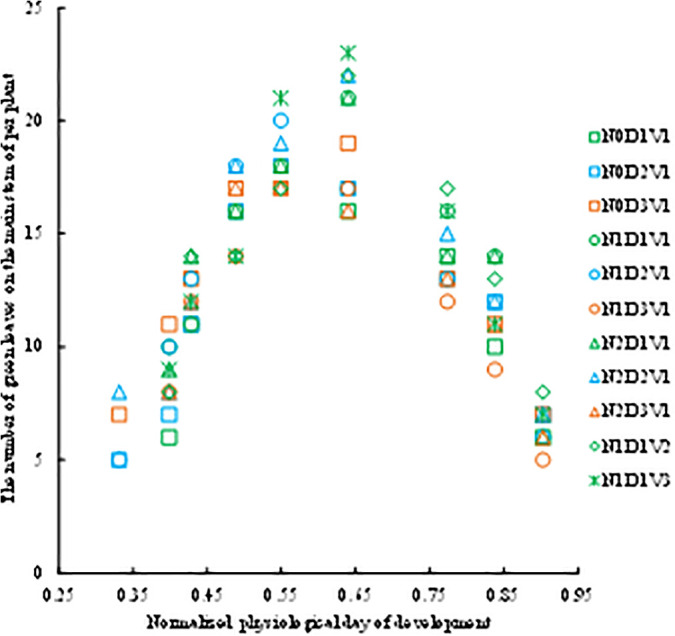
The changes in the green leaf numbers with normalized day of physiological development under different treatments in the 2013-2014 growing season.

With the continuous advancement of the growth process of rapeseed plants, every single leaf on the main stem also undergoes a process of elongation, continuation, and senescence. The experimental data showed that the green leaf numbers first increase and then decrease with increasing the normalized *DPD*, and reaching the maximum value when **n*DPD* is 0.64, that is, during the early anthesis stage (Fig 1) under the different treatments.

We analyzed the differences in leaf number and biomass partitioning coefficient under different treatments by using the multi-way ANOVA. The variance analysis result showed that the number of leaves is also affected by cultivar, cultivation measures (fertilization, transplanting density, etc.), and environmental conditions (Table 2).

**Table 2:** Variance analysis of the effects of growth stage, cultivar, and fertilizer, transplanting density, and its interactions on green leaf numbers (LN_Green_), and the leaf biomass partitioning coefficient of rapeseed main stem (PC_L_) at different growth stages. (2013-2014).

Source of variation	DF	LN_Green_	Significant for F	PC_L_	Significant for F
Growth stage (GS)	3	2444.029^***^	0.000	25062.376^***^	0.000
Nitrogen (N)	2	26.002^***^	0.000	557.815^***^	0.000
Transplanting density (TD)	2	17.414^***^	0.000	686.146^***^	0.000
GS × N	6	9.349^***^	0.000	335.675^***^	0.000
GS × TD	6	14.146^***^	0.000	237.866^***^	0.000
N × TD	4	17.621^***^	0.000	130.822^***^	0.000
GS × N × TD	12	4.044^***^	0.000	181.102^***^	0.000
Error	72				

“×” represents the interaction effect between indicators; ^*^, ^**^, and ^***^ indicate significant differences at the 0.05, 0.01, and 0.001 probability levels, respectively. The same as below.

### The main-stem leaf biomass partitioning coefficient model

#### The normalized leaf rank model.

**Fig 2:**
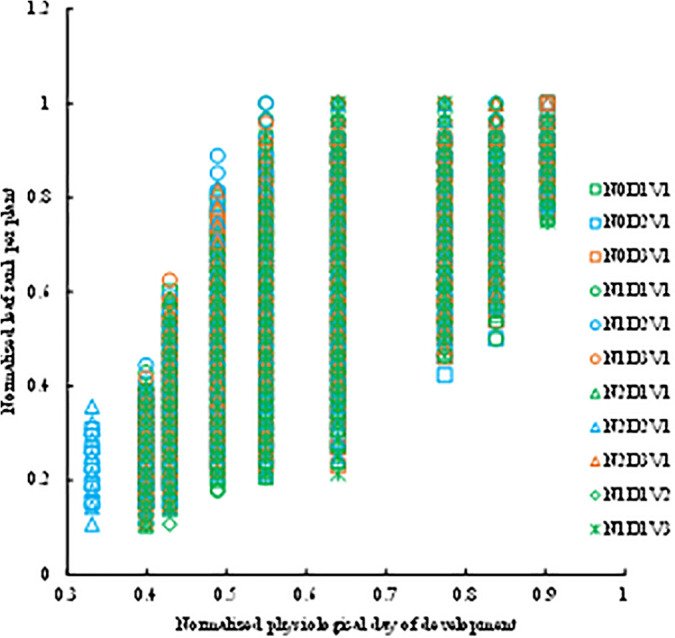
The changes in the normalized leaf rank with normalized day of physiological development under different treatments in the 2013-2014 growing season.


At any stage of rapeseed growth, there are varying numbers of green leaves on the main stem. However, even for the same cultivar grown in different years, the leaf count can vary, posing challenges for analyzing the change patterns of leaf biomass partitioning coefficients with *DPD*. To address this issue, we introduce the concept of normalized leaf rank (*nLR*) and proceed to investigate the relationship between normalized leaf rank and *DPD* variations. The experimental data showed that the values of *nLR*(*DPD*) of different treatments with normalized *DPD* were fitted by the logarithmic function (Fig 2, Equation 6).

$$nLR(DPD)=L_{{lr}_{\text{1}}}\text{ln}(nDPD)+L_{{lr}_{\text{2}}}$$ (6)

where nLR(DPD) is the normalized leaf rank at different normalized day of physiological development; nDPD is the normalized day of physiological development; $L_{{lr}_{\text{1}}}$, and $L_{{lr}_{\text{2}}}$ are model parameters. Detailed parameter estimation and significance test are shown in Table 3.

**Table 3:** The significant test of the main-stem leaf biomass partitioning coefficient models and their parameters.

*Eq.*	*n*	*r*	Significance level for *F*	Significant for *F*	Parameter	Estimate	Significance level for *t*	*t*
(6)	1167	0.691^***^	0.000	1061.941^***^	$L_{{lr}_{\text{1}}}$	0.618	0.000	32.587^***^
					$L_{{lr}_{\text{2}}}$	0.900	0.000	79.961^***^
(7)	11	0.990^***^	0.000	422.967^***^	$L_{{es}_{\text{1}}}$	1.836	0.000	20.566^***^
					$L_{{es}_{\text{2}}}$	−0.162	0.000	−10.295^***^
	11	0.961^***^	0.000	109.970^***^	$L_{{es}_{\text{3}}}$	−1.256	0.000	−10.487^***^
					$L_{{es}_{\text{4}}}$	0.506	0.000	13.584^***^
(8)	40	0.925^***^	0.000	225.694^***^	$L_{{ms}_{\text{1}}}$	1.480	0.000	15.023^***^
					$L_{{ms}_{\text{2}}}$	−0.131	0.000	−7.503^***^
	54	0.922^***^	0.000	144.166^***^	$L_{{ms}_{\text{3}}}$	3.426	0.000	4.397^***^
					$L_{{ms}_{\text{4}}}$	−3.314	0.000	−6.062^***^
					$L_{{ms}_{\text{5}}}$	0.826	0.000	8.784^***^
(9)	42	0.917^***^	0.000	210.436^***^	$L_{{ls}_{\text{1}}}$	1.060	0.000	14.506^***^
					$L_{{ls}_{\text{2}}}$	−0.100	0.000	−6.696^***^
	91	0.902^***^	0.000	388.571^***^	$L_{{ls}_{\text{3}}}$	1.562	0.000	5.638^***^
					$L_{{ls}_{\text{4}}}$	−7.998	0.000	−19.712^***^
(10)	74	0.875^***^	0.000	235.301^***^	$L_{{bu}_{\text{1}}}$	0.262	0.000	15.340^***^
					$L_{{bu}_{\text{2}}}$	1.000	0.000	12.814^***^
	101	0.925^***^	0.000	289.261^***^	$L_{{bu}_{\text{3}}}$	0.511	0.000	5.839^***^
					$L_{{bu}_{\text{4}}}$	−0.916	0.000	−8.182^***^
					$L_{{bu}_{\text{5}}}$	0.413	0.000	11.789^***^
(11)	88	0.961^***^	0.000	507.658^***^	$L_{{bo}_{\text{1}}}$	−0.012	0.912	−0.111
					$L_{{bo}_{\text{2}}}$	0.290	0.001	3.607^***^
					$L_{{bo}_{\text{3}}}$	−0.034	0.017	−2.439^*^
	119	0.961^***^	0.000	701.072	$L_{{bo}_{\text{4}}}$	0.605	0.000	10.969^***^
					$L_{{bo}_{\text{5}}}$	−1.130	0.000	−13.979^***^
					$L_{{bo}_{\text{6}}}$	0.531	0.000	18.321^***^
(12)	82	0.951^***^	0.000	762.519^***^	$L_{{eb}_{\text{1}}}$	0.498	0.000	12.862^***^
					$L_{{eb}_{\text{2}}}$	2.213	0.000	27.614^***^
	127	0.955^***^	0.000	643.546^***^	$L_{{eb}_{\text{3}}}$	0.971	0.000	13.063^***^
					$L_{{eb}_{\text{4}}}$	−1.792	0.000	−15.606^***^
					$L_{{eb}_{\text{5}}}$	0.827	0.000	19.044^***^
(13)	46	0.813^***^	0.000	85.532^***^	$L_{{fb}_{\text{1}}}$	0.342	0.000	9.248^***^
					$L_{{fb}_{\text{2}}}$	−0.067	0.001	−3.508^***^
	100	0.960^***^	0.000	569.971^***^	$L_{{fb}_{\text{3}}}$	0.870	0.000	9.096^***^
					$L_{{fb}_{\text{4}}}$	−1.713	0.000	−11.280^***^
					$L_{{fb}_{\text{5}}}$	0.848	0.000	14.278^***^
(14)	128	0.938^***^	0.000	459.989^***^	$L_{{lb}_{\text{1}}}$	0.053	0.622	0.495
					$L_{{lb}_{\text{2}}}$	−0.486	0.003	−2.978^**^
					$L_{{lb}_{\text{3}}}$	0.423	0.000	6.923^***^
(15)	78	0.899^***^	0.000	320.945^***^	$L_{m_{\text{1}}}$	−0.814	0.000	−17.915^***^
					$L_{m_{\text{2}}}$	0.024	0.003	3.106^**^

#### Model of leaf biomass partitioning coefficients for the main stem across growth stages.

**Leaf rank boundaries in the seedling stage: 0.10–0.63.** The seedling stage of winter rapeseed is longer, accounting for almost half or more of the whole growth period. The main-stem leaves are the largest nutrient organs at the seedling stage, and the biomass partitioning coefficient of rapeseed plants is mainly concentrated in the leaves. We divided the seedling stage of rapeseed into pre-winter stage, overwintering stage, and late seedling stage, which can also be called early seedling stage, mid-seedling stage, and late seedling stage. The leaf rank interval in the early seedling stage was the (0.10–0.24) and [0.24–0.38), and the leaf rank interval in the mid-seedling stage was the (0.10–0.25) and [0.25–0.50], and the late seedling stage was the (0.10–0.27) and [0.27–0.63).

**Fig 3:**
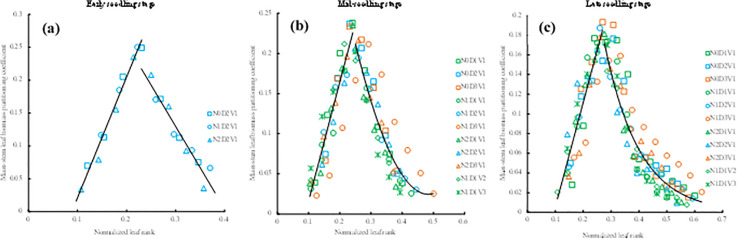
The changes in the main-stem leaf biomass partitioning coefficient with normalized leaf rank in the early seedling stage (a) and in the mid-seedling stage (b) and in the late seedling stage (c) under different treatments in the 2013-2014 growing season.


The main-stem leaf biomass partitioning coefficient of rapeseed in seedling stage under different treatments all showed a trend of increasing first and then decreasing (Fig 3). The values of *PC*_*ES*_(*LR*) of different treatments with normalized leaf rank were fitted by the linear function (Equation 7 and Fig 3a). The values of *PC*_*MS*_(*LR*) of different treatments with normalized leaf rank were fitted by a logarithmic function and a quadratic function (Equation 8 and Fig 3b). The values of *PC*_*LS*_(*LR*) of different treatments with normalized leaf rank were fitted by a linear function and a power function (Equation 9 and Fig 3c).

$${PC}_{ES}(LR)=\left\{ \begin{array}{c} L_{{es}_{\text{1}}}nLR+L_{{es}_{\text{2}}}\;\;\;\;\;\;\;\;(\text{0}.\text{1}\text{0}-\text{0}.\text{2}\text{4})\;\;\;\;\\ {L_{es}}_{\text{3}}nLR+L_{{es}_{\text{4}}}\;\;\;\;\;\;\;[\text{0}.\text{2}\text{4}-\text{0}.\text{3}\text{8})\;\;\;\; \end{array}\right.\;$$ (7)

$${PC}_{MS}(LR)=\left\{ \begin{array}{c} L_{{ms}_{\text{1}}}nLR+L_{{ms}_{\text{2}}}\;\;\;\;\;\;\;\;\;\;\;\;\;\;\;\;\;\;\;\;\;(\text{0}.\text{1}\text{0}-\text{0}.\text{2}\text{5})\;\;\;\\ L_{{ms}_{\text{3}}}{nLR}^{\text{2}}+L_{{ms}_{\text{4}}}nLR+L_{{ms}_{\text{5}}}\;\;\;[\text{0}.\text{2}\text{5}-\text{0}.\text{5}\text{0})\;\;\;\;\;\;\;\;\;\;\; \end{array}\right.\;$$ (8)

$${PC}_{LS}(LR)=\left\{ \begin{array}{c} L_{{ls}_{\text{1}}}nLR+L_{{ls}_{\text{2}}}\;\;\;\;\;\;\;\;\;(\text{0}.\text{1}\text{0}-\text{0}.\text{2}\text{7})\;\;\;\\ L_{{ls}_{\text{3}}}{nLR}^{L_{{ls}_{\text{4}}}}\;\;\;\;\;\;\;\;\;\;\;\;\;[\text{0}.\text{2}\text{7}-\text{0}.\text{6}\text{3})\;\;\; \end{array}\right.\;$$ (9)

where ${PC}_{ES}(LR)$, ${PC}_{MS}(LR)$, and ${PC}_{LS}(LR)$ are the leaf biomass partitioning coefficient at different leaf rank during the early stage, middle stage, and late stage of rapeseed seedling growth, respectively (g g^-1^); nLR(DPD) is the normalized leaf rank; $L_{{es}_{\text{1}}}$, $L_{{es}_{\text{2}}}$, $L_{{es}_{\text{3}}}$, $L_{{es}_{\text{4}}}$, $L_{{ms}_{\text{1}}}$, $L_{{ms}_{\text{2}}}$, $L_{{ms}_{\text{3}}}$, $L_{{ms}_{\text{4}}}$, $L_{{ms}_{\text{5}}}$, $L_{{ls}_{\text{1}}}$, $L_{{ls}_{\text{2}}}$, $L_{{ls}_{\text{3}}}$ and $L_{{ls}_{\text{4}}}$ are model parameters. Detailed parameter estimation and significance test are shown in Table 3.

**Leaf rank boundaries in budding and bolting stage: 0.17–1.0**. During the budding and bolting stage, there was a rapid accumulation of biomass in rapeseed leaves (Fig 1A). We divided the budding and bolting stage of rapeseed into budding stage, and bolting stage. The leaf rank interval in the budding stage was the (0.17–0.45) and [0.45–0.93), and the leaf rank interval in the bolting stage was the (0.21–0.50) and [0.50–1.0].

**Fig 4:**
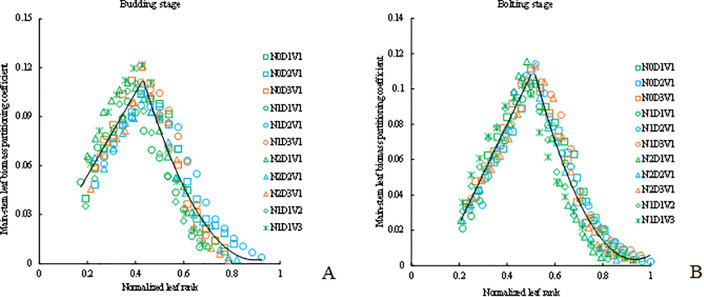
The changes in main-stem leaf biomass partitioning coefficient with normalized leaf rank in the budding stage (a) and in the bolting stage (b) under different treatments.


The main-stem leaf biomass partitioning coefficient of rapeseed in budding and bolting stage under different treatments all showed a trend of increasing first and then decreasing (Fig 4). The values of PC_BU_(*LR*) of different treatments with normalized leaf rank were fitted by a linear function and a quadratic function (Equation 10 and Fig 4a). The values of PC_BO_(*LR*) of different treatments with normalized leaf rank were both fitted by the quadratic function (Equation 11 and Fig 4b).

$${PC}_{BU}(LR)=\left\{ \begin{array}{cc} L_{{bu}_{\text{1}}}{nLR}^{L_{{bu}_{\text{2}}}} & (\text{0}.\text{1}\text{7}-\text{0}.\text{4}\text{5})\;\;\;\\ {L_{bu}}_{\text{3}}{nLR}^{\text{2}}+{L_{bu}}_{\text{4}}nLR+{L_{bu}}_{\text{5}} & [\text{0}.\text{4}\text{5}-\text{0}.\text{9}\text{3})\;\;\; \end{array}\right.\;$$ (10)

$${PC}_{BO}(LR)=\left\{ \begin{array}{cc} L_{{bo}_{\text{1}}}{nLR}^{\text{2}}+L_{{bo}_{\text{2}}}nLR+L_{{bo}_{\text{3}}} & (\text{0}.\text{2}\text{1}-\text{0}.\text{5}\text{0}\;\;\\ L_{{bo}_{\text{4}}}{nLR}^{\text{2}}+L_{{bo}_{\text{5}}}nLR+L_{{bo}_{\text{6}}} & (\text{0}.\text{5}\text{0}-\text{1}.\text{0}]\;\; \end{array}\right.\;$$ (11)

where ${PC}_{BU}(LR)$, and ${PC}_{BO}(LR)$ are the leaf biomass partitioning coefficient at different leaf rank during the budding stage and bolting stage, respectively (g g^-1^); nLR is the normalized leaf rank; $L_{{bu}_{\text{1}}}$, $L_{{bu}_{\text{2}}}$, $L_{{bu}_{\text{3}}}$, $L_{{bu}_{\text{4}}}$, $L_{{bu}_{\text{5}}}$, $L_{{bo}_{\text{1}}}$, $L_{{bo}_{\text{2}}}$, $L_{{bo}_{\text{3}}}$, $L_{{bo}_{\text{4}}}$, $L_{{bo}_{\text{5}}}$, and $L_{{bo}_{\text{6}}}$ are model parameters. Detailed parameter estimation and significance test are shown in Table 3.

**Fig 5:**
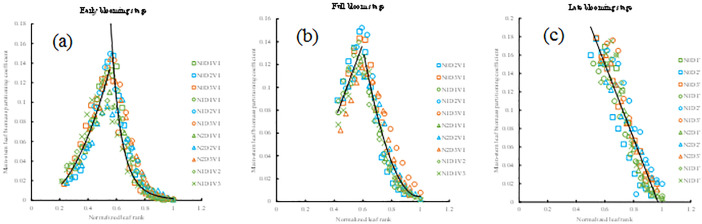
The changes in the main-stem leaf biomass partitioning coefficient with normalized leaf rank in the early blooming stage (a). in the full bloom stage (b), and in the late blooming stage (c) under different treatments.

**Leaf rank boundaries in anthesis and bear pods stage: 0.21–1.0.** The experimental data showed that the leaf number of rapeseed exhibits a decreasing trend during the anthesis stage, consequently leading to a decline in the biomass accumulation of the main stem. Conversely, the leaf biomass partitioning coefficient of rapeseed slightly increased from the early blooming stage to the full blooming stage and then to the late blooming stage as the total leaf number decreased (Fig 5). We divided the anthesis stage of rapeseed into the early blooming stage, full blooming stage, and late blooming stage. The leaf rank interval in the early blooming stage was the (0.21–0.55) and [0.55–1.0], and the leaf rank interval in the full blooming stage was the (0.42–0.60) and [0.60–1.0], and the late blooming stage was the [0.50–1.0].

The main-stem leaf biomass partitioning coefficient of rapeseed in early blooming stage under different treatments showed a trend of increasing first and then decreasing (Fig 5). The values of *PC*_*EB*_(*LR*) of different treatments with normalized leaf rank were fitted by a power function and a quadratic function (Equation 12 and Fig 5a). The values of *PC*_*FB*_(*LR*) of different treatments with normalized leaf rank were fitted by a linear function and a quadratic function (Equation 13 and Fig 5b). The values of *PC*_*LB*_(*LR*) of different treatments with normalized leaf rank were fitted by the quadratic function (Equation 14 and Fig 5c).


$${PC}_{EB}(LR)=\left\{ \begin{array}{cc} L_{{eb}_{\text{1}}}{nLR}^{L_{{eb}_{\text{2}}}} & (\text{0}.\text{2}\text{1}-\text{0}.\text{5}\text{5})\;\;\\ {L_{eb}}_{\text{3}}{nLR}^{\text{2}}+{L_{eb}}_{\text{4}}nLR+{L_{eb}}_{\text{5}} & [\text{0}.\text{5}\text{5}-\text{1}.\text{0}]\; \end{array}\right.\;$$ (12)

$${PC}_{FB}(LR)=\left\{ \begin{array}{cc} L_{{fb}_{\text{1}}}nLR+L_{{fb}_{\text{2}}} & (\text{0}.\text{4}\text{2}-\text{0}.\text{6}\text{0})\;\;\\ {L_{fb}}_{\text{3}}{nLR}^{\text{2}}+{L_{fb}}_{\text{4}}nLR+{L_{fb}}_{\text{5}} & [\text{0}.\text{6}\text{0}-\text{1}.\text{0}]\;\;\; \end{array}\;\;\;\;\;\;\right.\;$$ (13)

$${PC}_{LB}(LR)=L_{lb_{\text{1}}}{nLR}^{\text{2}}+L_{lb_{\text{2}}}nLR+L_{lb_{\text{3}}}\;\;\;\;\;\;\;[\text{0}.\text{5}\text{0}-\text{1}.\text{0}]$$ (14)

where${PC}_{EB}(LR)$, ${PC}_{FB}(LR)$ and ${PC}_{LB}(LR)$ are the leaf biomass partitioning coefficient at different leaf ranks during the early blooming stage, full blooming stage, and late blooming stage, respectively (g g^-1^); nLR is the normalized leaf rank; $L_{{eb}_{\text{1}}}$, $L_{{eb}_{\text{2}}}$, $L_{eb_{\text{3}}}$, $L_{{eb}_{\text{4}}}$, $L_{eb_{\text{5}}}$, $L_{{fb}_{\text{1}}}$, $L_{{fb}_{\text{2}}}$, $L_{{fb}_{\text{3}}}$, $L_{{fb}_{\text{4}}}$, $L_{{fb}_{\text{5}}}$, $L_{lb_{\text{1}}}$, $L_{lb_{\text{2}}}$ and $L_{{lb}_{\text{3}}}$ are model parameters. Detailed parameter estimation and significance test are shown in Table 3.

**Fig 6:**
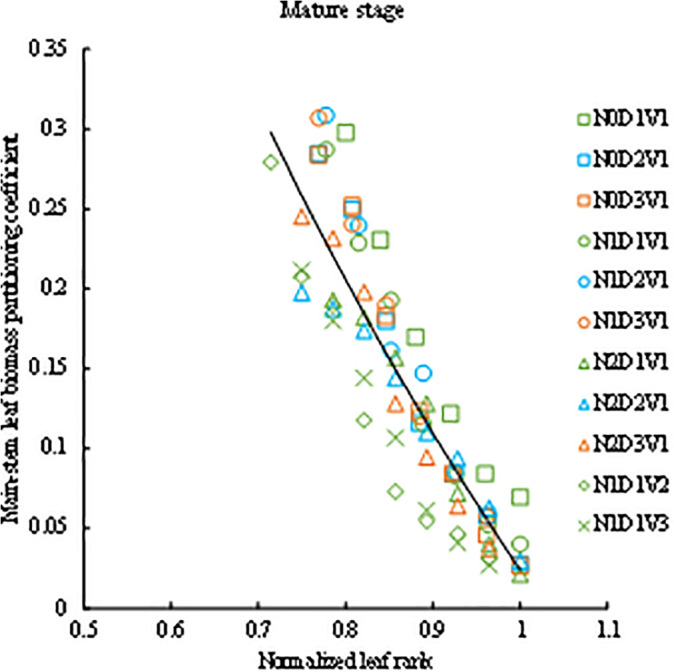
The changes in the main-stem leaf biomass partitioning coefficient with normalized leaf rank in the mature stage.

**Leaf rank boundaries in the mature stage: (0.71–1.0]**. During the mature stage, the biomass of rapeseed plants is primarily transferred to the siliques, with most of the main stem leaves initiating senescence and abscission (Fig 1A). Consequently, there is a significant reduction in leaf numbers, while the leaf biomass partitioning coefficient reaches its maximum values throughout the entire growth cycle (Fig 1B and Fig 6). The leaf rank interval in the mature stage was the (0.71–1.0].

The main-stem leaf biomass partitioning coefficient of rapeseed in the mature stage under different treatments all showed a decreasing trend (Fig 6). The values of *PC*_*M*_ (*LR*) of different treatments with normalized leaf rank were fitted by the logarithmic function (Equation 15 and Fig 6).


$${PC}_{M}(LR)=L_{m_{\text{1}}}\text{ln}(nLR)+L_{m_{\text{2}}}\;\;\;\;\;\;(\text{0}.\text{7}\text{1}-\text{1}.\text{0}]$$ (15)

where ${PC}_{M}(LR)$ is the leaf biomass partitioning coefficient at the different leaf ranks during the mature stage of rapeseed (g g^-1^); nLR is the normalized leaf rank; $L_{m_{\text{1}}}$ and $L_{m_{\text{2}}}$ are model parameters. Detailed parameter estimation and significance test are shown in Table 3.

### Model validation

**Fig 7:**
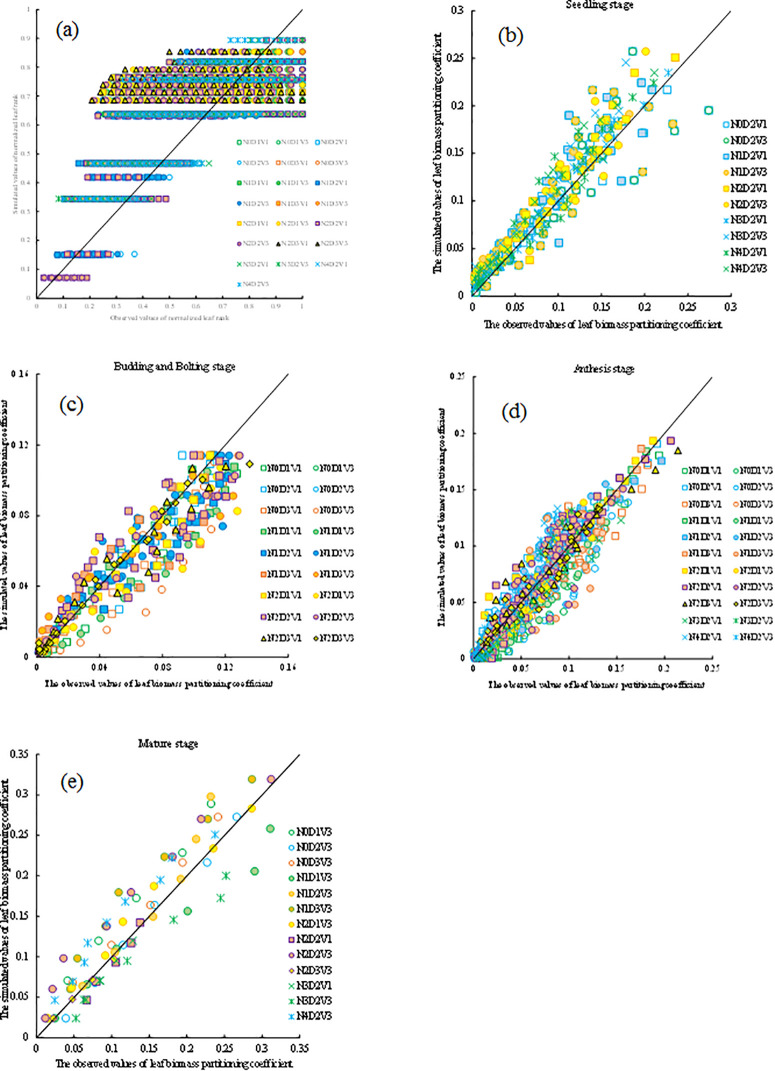
Comparison of the main-stem average leaf biomass partitioning coefficient (a), the leaf biomass partitioning coefficient observed values with the simulated values at (b) the seedling stage, (c) the budding and bolting stage, (d) the anthesis stage, and (e) the mature stage under different treatments in the 2012-2013 and 2014-2015.

The model developed in this paper is validated using independent experimental data in the 2012−2013 and 2014−2015, and the results showed that the correlation (*r*) of simulation and observation values for the average leaf biomass partitioning coefficient and the leaf biomass partitioning coefficient under different treatments during different growth stages all had a significant level at *p* < 0.001. The absolute values of the average absolute difference (*d*_*a*_) are all less than 0.011 g g^-1^, the values of the root mean square error (*RMSE*) are all less than 0.193 g g^-1^, the ratio of *d*_*a*_ to the average observation (*d*_*ap*_) are all less than 13.083%, which indicated that the observed and simulated leaf biomass partitioning coefficient were all close to the 1:1 line. The average absolute difference (*d*_*a*_) values for normalized leaf rank (*nLR*), leaf biomass partitioning coefficient at the seedling stage (*PCs*), and at the budding and bolting stage (*PC*_*Bb*_), and at the anthesis stage (*PC*_*A*_), and at the mature stage (*PC*_*M*_) under different treatments are −0.080 g g^-1^, −0.010 g g^-1^, 0.007 g g^-1^, 0.002 g g^-1^, and −0.011 g g^-1^, respectively. The root mean square error (*RMSE*) values for *nLR*, *PCs*, *PC*_*Bb*_, *PC*_*A*_, and *PC*_*M*_ are 0.193 g g^-1^, 0.032 g g^-1^, 0.016 g g^-1^, 0.017 g g^-1^, and 0.032 g g^-1^, respectively. The ratio of *d*_*a*_ to the average observation (*d*_*ap*_) of simulation and observation values for *PC*_*B*_ is less than 5%, the *PC*_*M*_ is less than 10%, the PCs and *PC*_*Bb*_ are less than 12%, which indicate that the models constructed in this study have a good performance and reliability (Fig 7 and Table 4).

**Table 4:** Comparison of statistical parameters of observation and simulation in the main-stem leaf biomass partitioning coefficient models in the 2012-2013 and 2014-2015.

Architectural parameter	Statistic parameters of simulation and observation				
	*n*	*d*_*a*_ (g g^-1^)	*d*_*ap*_ (%)	*RMSE* (g g^-1^)	*r*
Normalized leaf rank (nLR)	1840	−0.080	13.083	0.193	0.657^***^
Leaf biomass partitioning coefficient at seedling stage (PCs)	291	−0.010	11.236	0.032	0.943^***^
Leaf biomass partitioning coefficient at budding and bolting stage (PC_Bb_)	381	0.007	11.312	0.016	0.936^***^
Leaf biomass partitioning coefficient at anthesis stage (PC_A_)	775	0.002	3.077	0.017	0.936^***^
Leaf biomass partitioning coefficient at mature stage (PC_M_)	84	−0.011	8.661	0.032	0.938^***^

## Discussion

### Effects of nitrogen fertilization on leaf biomass partitioning in rapeseed

The vegetative phase in rapeseed establishes the photosynthetic infrastructure critical for yield formation, with leaf biomass accumulation serving as both a driver and indicator of reproductive potential [24]. Nitrogen (N) fertilization significantly enhances photosynthetic performance by increasing ribulose-1,5-bisphosphate carboxylase/oxygenase (RuBisCO) activity and leaf area index (LAI), thereby improving canopy light interception efficiency [25,26]. Conversely, N-deficient conditions induce leaf structural constraints, including reduced mesophyll cell density and impaired stomatal conductance, which constrain light harvesting and carbon fixation, creating metabolic bottlenecks during early developmental stages [27,28].

During reproductive transition, leaves undergo functional specialization from autonomous carbon sources to transient sinks, a shift modulated by silique-dominated assimilate competition [29–32]. This sink hierarchy precipitates architectural trade-offs, as developing reproductive structures shade lower leaves, reducing their photosynthetic active radiation (PAR) interception and accelerating senescence through cytokinin redistribution [33–35]. This architectural trade-off is genotype-dependent, with cultivars exhibiting differential strategies in maintaining source-sink balance through flowering progression [36,37]. Leaf size and plant numbers during this phase serve as key predictors of subsequent yield components.

Adequate nitrogen supply plays a crucial role in shaping biomass allocation patterns within rapeseed plants, particularly in their leaves. Optimal nitrogen levels promote growth and development, resulting in increased leaf biomass. This increase in leaf biomass is often accompanied by changes in biomass allocation, with a higher proportion of total biomass being allocated to leaves compared to other plant organs [38]. Moreover, the application of nitrogen fertilizer can influence the structural and physiological characteristics of rapeseed leaves. It can lead to larger leaf area, higher chlorophyll content, and improved photosynthetic activity. These alterations in leaf morphology and physiology contribute to enhanced carbon assimilation and ultimately higher biomass production in rapeseed plants [39]. However, it is important to note that excessive nitrogen application can have negative effects on biomass allocation in rapeseed leaves. It can disrupt nutrient uptake balance, heightened susceptibility to diseases, and cause environmental stress. Furthermore, nitrogen oversupply can promote vegetative growth at the expense of reproductive development, potentially compromising seed yield and quality [40].

### Effects of plant density on leaf biomass partitioning in rapeseed

Planting density represents a critical agronomic factor impacting crop productivity and resource use efficiency, with significant implications for leaf biomass partitioning patterns in rapeseed [41,42]. Studies have indicated that rapeseed plants cultivated at higher densities generally exhibit accelerated growth rates and enhanced biomass accumulation compared to those at lower densities [30,43]. Nevertheless, the complex mechanisms through which transplanting density modulates biomass partitioning in rapeseed leaves necessitate more in-depth exploration.

Elevated planting densities typically intensify inter-plant competition for limited resources such as light, water, and nutrients. As an adaptive strategy, rapeseed plants often modify their biomass allocation patterns, particularly in leaf organs, to cope with intensified competitive pressures. For instance, Kuai *et al*. revealed that increasing planting density induced notable shifts in biomass partitioning, where lower densities promoted a higher proportion of biomass allocated to leaves [44]. Concurrently, higher-density plantings were associated with the development of taller and more elongated plant architectures, accompanied by increased Leaf Area Index (LAI) and Radiation Use Efficiency (RUE) across successive growth stages [45]. These observations indicate that planting density not only regulates biomass distribution but also influences plant morphological traits and resource utilization efficiency.

Understanding how planting density affects rapeseed leaf growth is crucial for elucidating the implications for biomass accumulation and distribution along the main stem [46]. Experimental evidence demonstrates that varying planting densities result in distinct patterns of leaf biomass partitioning: higher densities tend to reduce biomass allocation to lower leaf ranks while promoting greater accumulation in upper leaf positions [43,47]. In contrast, lower planting densities facilitate more equitable biomass distribution along the main stem, likely due to diminished inter-plant competition for essential resources. This rank‑specific response underscores the importance of considering vertical leaf heterogeneity in biomass allocation models. Recent work on cucumber has similarly highlighted the value of leaf‑position‑specific photosynthetic modelling, showing that such an approach significantly improves the accuracy of dry‑matter partitioning predictions compared to uniform canopy representations [11]. Although rapeseed differs morphologically and physiologically from cucumber, the principle that leaf rank mediates both photosynthetic capacity and assimilate allocation appears to be a general feature of canopy function, supporting the relevance of our rank‑based partitioning framework.

During the vegetative growth stage, leaves serve as primary organs for biomass accumulation, playing a pivotal role in establishing the foundation for subsequent grain formation [24,48]. Adequate biomass accumulation during this stage is therefore critical for achieving optimal grain yield in later developmental stages.

### Mechanistic modelling of biomass accumulation and distribution in rapeseed

The quantitative prediction of biomass allocation patterns has emerged as a critical frontier in rapeseed yield, with researchers worldwide employing diverse modelling approaches to dissect these processes. Notable models include the DAR95 (DAISY-Rape) model developed by Petersen *et al*. [49], which utilizes the distribution coefficient method to illustrate biomass allocation to different plant organs, including seeds. Additionally, Gabrielle et al. [50] developed a CERES-type model for winter oilseed rape that integrated environmental factors such as nitrogen availability into biomass allocation simulations, addressing the limitation of DAR95 in ignoring abiotic factor impacts on organ growth. Habekotté [51] further optimized crop growth modelling by evaluating key seed yield-determining factors through simulations, which laid a foundation for linking biomass distribution to actual yield formation rather than just organ growth. Moreover, the LINTUL-BRASNAP model, a light interception and utilization simulator, quantified the relationship between canopy light absorption efficiency and biomass accumulation dynamics, enriching the mechanistic understanding of source-sink relationships in rapeseed by linking resource capture to dry matter partitioning. Liu *et al*. and Wang *et al*. further advanced this field by introducing dynamic simulation models that link aboveground allocation indices to physiological developmental time, offering temporal insights into biomass redistribution [52,53]. Tang *et al*. explored cultivar- and nitrogen fertilizer-driven variations in biomass distribution, though their model’s generality could be enhanced by integrating additional environmental variables such as temperature and light intensity [54]. Building on these foundations, Cao *et al*. developed a mechanistic model incorporating photosynthetic processes and microclimatic factors to simulate rapeseed growth dynamics [21]. Robertson *et al*. applied APSIM platform to create APSIM-Canola, a comprehensive model detailing phenological and morphological development [55]. Conversely, the EPR95 (EPIC-Rape) model by Kiniry *et al*. offers broad applicability across crops and environments but relies on effective accumulated temperature, a limitation that may compromise its accuracy in predicting rapeseed development under heterogeneous climatic conditions despite its user-friendly interface [56].

Current challenges in biomass modelling necessitate the development of more integrative frameworks that capture the dynamic interplay between genetic, physiological, and environmental drivers of biomass partitioning. Future research should prioritize mechanistic models capable of resolving how planting density, nutrient availability, and climatic variables modulate allocation patterns across vegetative (e.g., leaves, stems) and reproductive organs. For instance, refining leaf biomass partitioning coefficient models, such as those developed herein, can enhance understanding of vertical biomass distribution along the main stem, a critical factor in optimizing radiation interception and resource use efficiency.

The present study builds on prior characterizations of biomass allocation across organs (e.g., main stem, total leaves, and branches) by introducing a hierarchical model of leaf rank-specific biomass partitioning. This approach addresses a key gap in existing literature by dissociating biomass allocation dynamics among upper and lower leaf ranks, which are differentially influenced by light competition and source-sink relationships. In this study, the validation results confirm the model reliably simulates leaf biomass partitioning across key developmental stages. While the model demonstrates robust overall performance, elevated *d*_*ap*_ values (>10%) occur specifically during seedling establishment (PCs), stem elongation (PC_Bb_), and across normalized leaf ranks (*nLR*). We attribute this phenomenon to two interrelated factors characteristic of rapeseed’s vegetative growth phase: First, inherently low biomass partitioning coefficients during rapid leaf expansion amplify the relative impact of minor absolute errors. Second, subtle environmental variations (such as microclimate gradients or resource availability), induce substantial biological variability in foliar development patterns, particularly at individual leaf positions. These combined effects naturally increase dispersion between observed and simulated values during these dynamic growth stages.

By integrating empirical data with process-based algorithms, these models not only advance theoretical understanding of rapeseed functional ecology but also provide actionable tools for precision agriculture, enabling farmers to optimize planting density and nitrogen management to enhance yield potential. Prospective validation under diverse field conditions will be essential to refining model parameters and ensuring their scalability. Ultimately, such advancements in functional-structural modelling will strengthen our capacity to predict crop performance under climate change scenarios, fostering the development of resilient and resource-efficient rapeseed production systems.

## Conclusions

This study developed and validated a leaf biomass partitioning coefficient model for the main stem of rapeseed across different leaf ranks. The model was established using three years of partial field experimental data encompassing three cultivars, five nitrogen levels, and three planting densities. By analyzing the quantitative relationship between the leaf biomass partitioning coefficient and the normalized leaf rank (*nLR*), we established stage-specific descriptive models for the seedling, budding-bolting, anthesis, and mature growth phases.

Validation with independent experimental data demonstrated the model’s robustness. The simulated values showed statistically significant correlations with the observed data (*r* > 0.9, *p* < 0.001). The mean absolute difference (*d*_*a*_) ranged from −0.080 to 0.007 g g^-1^, and the ratio of *d*_*a*_ to the average observation (*d*_*ap*_) varied between 3.077% (anthesis stage) and 13.083% (normalized leaf rank). The root mean square error (*RMSE)* fell below 0.193 g g^-1^ across all stages, with the most stage-specific *RMSE* values under 0.032 g g^-1^.

Collectively, these results demonstrate that the model reliably simulates the biomass partitioning coefficient of the main‑stem leaves across hierarchical leaf ranks in rapeseed. The work provides a quantitative tool for analyzing source-sink allocation patterns and offers a foundation for further integration of rank‑specific leaf dynamics into functional–structural crop models.

## Supporting information

S1 Table The raw values underlying the statistical analyses presented in the manuscript (Table 2). (XLSX)

S2 Table The main-stem leaf biomass partitioning coefficient model to generate Figs 1–6 and Table 3. (XLSX)

S3 Table The raw data for model validation results Fig 7 and Table 4. (XLSX)

S1 File Appendix. (DOCX)
