# Supplementary material for: Model of leaf biomass partitioning coefficient in different main stem leaf ranks of rapeseed (Brassica napus L.)
Source: PLoS One. 2026 Feb 5;21(2):e0330011. doi: 10.1371/journal.pone.0330011 (PMC12875455; doi:10.1371/journal.pone.0330011)
Supplement: S1 File — (DOCX) [file pone.0330011.s001.docx]

Appendix A. Day of physiological development (*DPD*), which was used to drive the model [20], is defined as follows

*DPD =*$\sum_{i=1}^{N} e^{kj}.{T_{ebj}}^{pj}.{T_{euj}}^{qj}.{P_{ej}}^{Gj}.f(E_{Ci})$ Equation (A.1)

*T_ebj_* = (*T_i_* − *T_bj_*)/(*T_oj_* − *T_bj_*), *T _i_* = *T _bj_*, when *T_i_* <*T _bj_*; *T_i_* = *T_oj_*, when *T_i_* >*T_oj_*.*T_euj_* = (*T_uj_* − *T_i_*)/(*T_uj_* − *T_oj_*), *T_i_* = *T_uj_*, when *T_i_* >*T_uj_*.*P_ej_* = (*P_i_* − *P_bj_*)/(*P_oj_* − *P_bj_*), *P_i_* = *P_bj_*, when *P_i_* <*P_bj_*; *P_i_* = *P_oj_*, when *P_i_* >*P_oj_*.

where the *N* is the number of days covering the stage, the *k_j_* is a basic development parameter which is determined by cultivar heredity, the *T_ebj_* and the *T_euj_* are the effective factors for lower and upper temperature, respectively, the *pj* and the *q_j_* are the genotypic coefficients of temperature effects, the *P_ej_* is the effective factor of photoperiod, *G_j_* is the genotypic coefficient of photoperiod effects, and f (*E_Ci_*) is the effective function of agronomic practice factors for rapeseed. *T_i_* is the daily mean temperature ($℃$) in the *j*th stage, *T_bj_*, *T_oj_* and *T_uj_* are lower, optimum, and upper limit temperature ($℃$) demanded in the *j*th stage for rapeseed, respectively, and *P_bj_*, *P_oj_* are the critical and optimum day length (h) demanded in the *j*th stage for rapeseed, respectively. *DPD* = 0, 1, 2, 3, and 4 represent sowing, seedling, bolting, flowering, and maturity, respectively.
